# HIV risk screening and HIV testing among orphans and vulnerable children in community settings in Tanzania: Acceptability and fidelity to lay-cadre administration of the screening tool

**DOI:** 10.1371/journal.pone.0248751

**Published:** 2021-03-25

**Authors:** Michelle M. Gill, Ola Jahanpour, Roland van de Ven, Asheri Barankena, Peris Urasa, Gretchen Antelman

**Affiliations:** 1 Elizabeth Glaser Pediatric AIDS Foundation, Washington, DC, United States of America; 2 Elizabeth Glaser Pediatric AIDS Foundation, Dar es Salaam, Tanzania; 3 Department of Epidemiology and Biostatistics, The Institute of Public Health, Kilimanjaro Christian Medical University College, Moshi, Tanzania; 4 Pact, Dar es Salaam, Tanzania; 5 Ministry of Health, Community Development, Gender, Elderly and Children, Dodoma, Tanzania; University College London, UNITED KINGDOM

## Abstract

**Introduction:**

HIV risk screening tool validation studies have not typically included process evaluations to understand tool implementation. The study aim was to assess the fidelity to which an HIV risk screening tool was administered by lay workers and acceptability of delivering home-based screening coupled with HIV testing to beneficiaries in an orphans and vulnerable children (OVC) program.

**Methods:**

This cross-sectional study was conducted March-April 2019 in two regions of Tanzania. Community case workers (CCW) were observed conducting screenings with OVC 2–19 years and participated in focus group discussions. Research staff used structured observation checklists to capture if screening questions were asked or reworded by CCW. In-depth interviews were conducted with older adolescents and caregivers in their homes following screening and testing. A composite score was developed for the checklist. Qualitative data were thematically analyzed to address screening and testing perceptions and experiences.

**Results:**

CCW (n = 32) participated in 166 observations. Commonly skipped items were malnutrition (34% of all observed screenings) and sexual activity and pregnancy (20% and 45% of screenings for adolescents only). Items frequently re-worded included child abuse (22%) and malnutrition (15%). CCW had an average composite observation score of 42/50. CCW in focus groups (n = 34) found the screening process acceptable. However, they described rewording some questions viewed as harsh or socially inappropriate to ask. Overall, adolescent beneficiaries (n = 17) and caregivers (n = 25) were satisfied with home-based screening and testing and reported no negative consequences. Learning one’s HIV negative status was seen as an opportunity to discuss or recommit to healthy behaviors. While respondents identified multiple benefits of home testing, they noted the potential for privacy breaches in household settings.

**Conclusions:**

We found sub-optimal fidelity to the administration of the screening tool by CCW in home environments to children and adolescents enrolled in an OVC program. Improvements to questions and their delivery and ongoing mentorship could strengthen tool performance and HIV case finding using a targeted testing approach. Overall, home-based HIV risk screening and testing were acceptable to beneficiaries and CCW, could improve testing uptake, and serve as a platform to promote healthy behaviors for those with limited health system interactions.

## Introduction

While gains have been made in HIV testing in recent years, globally we have fallen short of reaching the first UNAIDS target, 90% of people living with HIV diagnosed by 2020, and are now aiming to surpass this goal and reach 95% by 2030 [1]. In 2018, 79% [67–92%] of all people living with HIV knew their HIV status [2]. Reaching this target is an even greater challenge among pediatric and adolescent populations. In eastern and southern Africa, 90% of women and 80% of men aged 25 and above knew their HIV status, while only 66% and 50% of young women and men aged 15–24, respectively, knew their status [3]. In Tanzania, nearly two-thirds (64%) of 15- to 19-year-old adolescents living with HIV were not aware of their HIV status and half (50%) of children 0–14 years living with HIV were undiagnosed [4]. Tanzania has relatively low HIV prevalence among children and adolescents. In 2018, HIV prevalence in the country among children was 0.4% and 1.4% for adolescents 15–24 years [4].

Barriers to HIV testing include lack of youth-friendly services, poor attitudes of health providers, fear of stigmatization and other negative consequences of a positive diagnosis, and age thresholds for minor consent for HIV testing [5–7]. There are also considerable gaps in early infant diagnostic testing, with only 52% of HIV-exposed infants tested by two months of age in 23 high burden countries largely in sub-Saharan Africa [8]. Once the opportunity to test as part of the prevention of mother-to-child HIV transmission program has been missed, other HIV testing approaches to identify children living with HIV include routine testing of children admitted to the hospital, targeted testing of siblings and biological children of patients enrolled in HIV care, community-based testing, and integration with immunization and other services [9, 10]. However, it has become increasingly more difficult to identify individuals with undiagnosed HIV infection in resource-constrained settings and in an uncertain funding environment [11–13]. HIV risk screening tools have been promoted as one approach to make more efficient use of resources and improve HIV positivity yield, particularly among low HIV prevalence populations. Initial efforts to develop a screening tool-based approach to identify children and adolescents for HIV testing were largely conducted at the health facility level through clinical symptom screening by medical personnel [14–18]. However, this facility-based approach does not specifically target orphans and vulnerable children (OVC) and excludes children and adolescents served by community-based programs.

Providing HIV testing services (HTS) to OVC in their homes or orphanages in a moderate and high-prevalence setting yielded an HIV positivity rate of 2.6% and 0.4% respectively; in one study it was the first HIV test that half of the orphans ≤ 17 years had ever received [19–21]. Home-based approaches may be able to address some of the HIV testing barriers at the facility level, such as those related to access and confidentiality [9]. However, other barriers, such as fear of stigma surrounding a possible HIV positive diagnosis would persist and home-based delivery of HIV services may present additional challenges [5, 7, 9].

HIV risk screening tool validation studies in children have focused on identifying a set of screening items to optimize the sensitivity, specificity, and predictive value [16, 17, 22]. Having a highly sensitive screening tool that correctly identifies children at risk for HIV and prompts HIV testing will minimize the number of children living with HIV who would be missed. While screening items may be less sensitive if they do not accurately reflect risk factors, items may also be skipped or otherwise not asked in a manner that yields an accurate response. An assessment of the fidelity of tool implementation should be integrated into validation studies, particularly as there is expanded use of these tools in community-based settings and as part of task shifting to lay cadres.

While there is little published evidence on the extent to which screening questions are asked with fidelity, there are also limited data on the overall process of HIV risk screening and HIV testing, particularly when targeted to OVC in their home environments. The aim of this study was to assess the fidelity to which an HIV risk screening tool was administered by a lay cadre and the acceptability of home-based screening and testing in an OVC program. Observational and qualitative data were collected to help contextualize the community-based screening model for OVC program beneficiaries and complement the findings of the main study on tool validation.

## Methods

### Project and main study description

**Fig 1:**
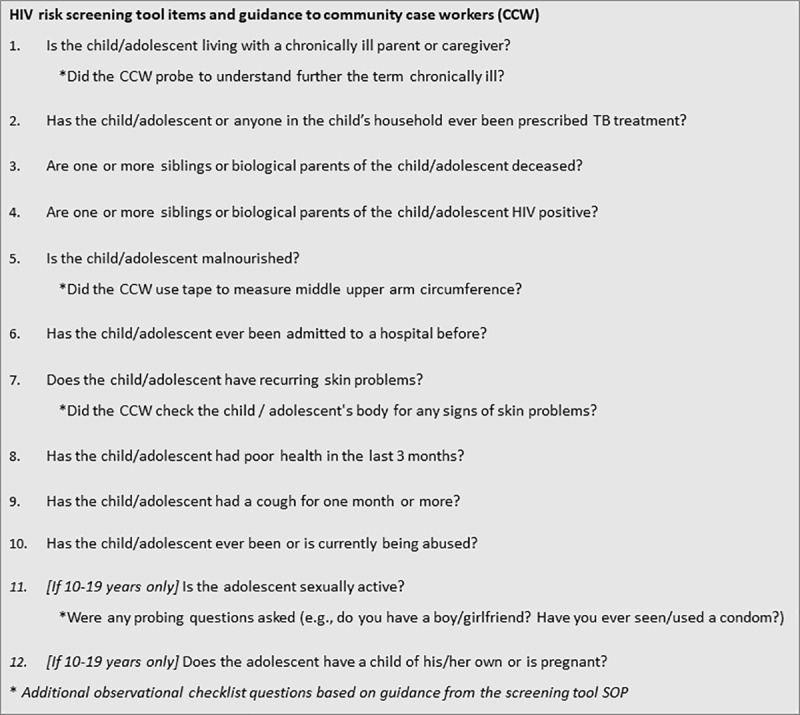
HIV risk screening tool items and guidance to Community Case Workers (CCW).

This cross-sectional study was conducted as part of a larger study evaluating the performance of an HIV risk screening tool administered to beneficiaries of an OVC program. Households were registered in the program if they met certain risk criteria, including being in a household headed by a child, having one or more single or double orphans, having a household member that is living with HIV, having a child in the household who is severely malnourished, and/or having a caregiver who is a sex worker. HIV risk screening was conducted during program enrollment of child and adolescent beneficiaries 2–19 years of age in registered households. The screening tool was administered by community case workers (CCW) supporting the OVC program, who were employed by local civil society organizations (CSO). The tool included 12 questions (Fig 1), comprised of validated risk factors from the literature and other items describing individual- or family-level HIV-related vulnerabilities [17]. CCW typically determined who would be involved in the screening, based on each household’s unique situation and beneficiary/caregiver preferences. In general, CCW administered the tool to caregivers of children < 10 years and to adolescents ≥ 10 years, with or without their caregivers present. Under the program, beneficiaries who provided positive answers to one or more items on the tool were referred for facility-based HIV testing, in addition to any referrals for other medical or social services.

For the main study activities conducted in community settings in February-April 2019, CCW were accompanied by research nurses who were responsible for recruitment, the enrollment interview, and HIV testing, in accordance with national guidelines. Using the responses from the CCW-administered HIV risk screening tool and the HIV testing outcomes, the primary aim of the overall study was to validate the 12-item tool. Children/adolescents age 2–19 years were eligble for the main study if they were registered in the program (using the risk criteria described above) in a study region in Tanzania and excluded if they had a history of antiretroviral therapy, were <5 years and tested HIV-negative after breastfeeding cessation, or were ≥ 5 years and tested HIV-negative in the past 6 months with no reported HIV exposure since their last HIV test.

### Data collection

Data collection for the sub-study took place March-April 2019 in two wards each in the Dar es Salaam and Tabora regions. For a subset of households, a research assistant (RA) accompanied nurse/CCW teams and was responsible for observing the risk screening process and conducting in-depth interviews (IDI) following home-based screening and testing. RAs observed the CCW-administered HIV risk screening and aspects of the home environment using a structured checklist. RAs documented whether or not a question was asked and, if it was asked, whether or not it was reworded (yes, no, partly).

Additional checklist questions captured whether or not CCW followed guidance detailed in the standard operating procedures (SOP) for tool use (noted with an asterisk in Fig 1) and general communication skills, such as securing a private area, asking questions in a non-judgmental manner, and addressing questions or concerns raised during the screening. RAs also included their own comments to help explain the structured responses and to capture CCW explanations as applicable. CCW provided written informed consent to participate in one or more observations. Adolescents (≥ 10 years) and caregivers of children and non-emancipated adolescents 2–17 years provided verbal permission to be observed, obtained through use of an institutional review board (IRB)-approved information sheet administered and signed off on by RAs. Beneficiary answers to the screening questions and other personal information were not recorded as part of observations.

After the observations, other activities for the main study took place, including HIV testing and counseling by the research nurse. RAs then recruited caregivers of non-emancipated children and adolescents 2–17 years and older adolescents (18–19 years and emancipated minors 15–17 years) who could consent for themselves, to participate in IDI. Forty-two IDI were conducted among approximately 1,400 households from the two regions participating in the main study. We aimed for roughly equal targets of caregivers with children 2–9 years, caregivers of adolescents 10–17 years, and older adolescents. Because caregivers often had multiple children in their care, many contributed to the enrollment target for both age groups (2–9 and 10–17 years). Inclusion criteria for IDI participants were enrollment into the main study’s community data collection activities and completion of the HIV risk screening tool. We attempted to interview beneficiaries who refused to participate in the home HIV testing, but there were no such refusals during the data collection period. Adolescents and caregivers provided oral informed consent prior to study participation. Interviews took place at the same visit as the main study activities or during a follow-up visit. Most interviews were completed in approximately 30 minutes or less. The majority of interviews were conducted in the home, with a few others conducted outside or in another location if there was the potential for privacy breach in the household. The IDI guide captured perceptions and experiences with home-based screening and testing, their ease in answering screening questions, and preferences for the location of HIV testing services. Close-ended questions were included on demographics and screening/testing acceptability.

Separately, CCW who had worked for the CSO for ≥6 months, who were responsible for HIV risk screening, and who were involved in household visits where HIV testing had taken place as part of the study were recruited to participate in four focus group discussions (FGD). Two FGD each were conducted in the Dar es Salaam and Tabora regions at a local Government office or hospital. FGD were comprised of 8–9 CCW each and lasted approximatley one and a half hours. All CCW participating in the FGD provided written informed consent and were reimbursed for their transport to the venue. The qualitative guide included questions about their perspectives on the content of the screening tool and its ease of use, their experience with the screening process, and HIV testing in the home and through facility referral.

### Data preparation and analysis

All IDI and FGD were audio recorded and conducted in Kiswahili. Recordings were transcribed and translated into English transcripts. Two additional study staff members fluent in Kiswahili and English verified all transcripts against the original audio recordings to ensure that the transcriptions and translations were accurate. Transcripts were imported into a qualitative software program for management (Atlas V7.1). A preliminary *a priori* codebook was developed with deductive codes based on the qualitative guides. Four coders trained in qualitative analysis coded the same transcripts, compared the assigned codes for similar text segments, and resolved any discrepancies, sometimes through refining or introducing new codes, until consensus was reached. Once the final set of codes were agreed upon, the four coders coded the remaining transcripts individually. Data were analyzed using a thematic analysis approach by participant category: caregivers of children by age group, adolescents, and CCW. This involved careful reading of transcripts to identify recurrent patterns and themes, reducing data into matrices and summaries, and drawing conclusions from issues connected to the study objectives. The quantitative data on participant demographics and acceptability statements collected as part of the IDI and FGD were entered into an Excel database and summarized descriptively.

Data from the structured observations were analyzed descriptively, with summary statistics calculated for the close-ended questions related to observations of CCW administration of the screening items using frequencies and percentages for categorical variables and medians and interquartile ranges for continuous variables. The open-ended comments from the observers were organized by screening item and used to contextualize the quantitative data. A composite score was developed which coded screening items on a 3-point scale: item asked and not reworded (3 points), item asked and partly reworded (2 points), item asked and reworded (1 point), item not asked (0 points). Scores were adjusted based on age group, as not all questions were intended for all beneficiaries. Additional questions on SOPs and general communication were calculated separately, but included as a part of the overall score.

### Ethical approval

The protocol was approved by the National Research Ethics Committee of the National Institute for Medical Research in Tanzania and the Population Council IRB based in the USA.

## Results

### Demographics

Table 1 presents the number of CCW observations and caregiver/adolescent IDI by the age group of the children involved and by region. CCW (n = 32, 16 in each region) were observed administering the HIV risk screening tool to beneficiaries 1–14 times each for a total of 166 observations. Of the 42 IDI conducted, 25 interviews (59.5%) were with caregivers. Six caregivers had children 2–9 years of age, six were caregivers of children 10–17 years, and 13 caregivers were caring for children in both age groups. Of the 17 adolescent IDIs, only one emancipated minor age 16 years participated in an interview; the remaining adolescents were 18 or 19 years of age. All IDI participants accepted child/adolescent screening and testing services and no child/adolescent tested HIV-positive in our sample.

**Table 1:** Number of observations and IDI conducted by child age and region.

Type of participant	Observations of CCW			Caregiver and adolescent IDI		
	Tabora	Dar es Salaam	Total	Tabora	Dar es Salaam	Total
**Caregivers of children 2–9 years**	48	34	82	2	4	6
**Caregivers of adolescents 10–17 years**	46	18	64	2	4	6
**Caregivers of children / adolescents in both age groups**	--	--	--	10	3	13
**Emancipated minors (15–17) or adolescents 18–19 years**	12	8	20	10	7	17
**TOTAL**	106	60	166	24	18	42

Among 34 FGD participants, median respondent age was 38.0 years (interquartile range [IQR] 31.3–46.0); 47.1% were female. Nineteen (55.9%) CCW had completed primary school and 15 (44.1%) had some secondary education or more. CCW had been conducting OVC household visits for a median of one year (IQR 1.0–2.0). Some CCW participated in both the FGD and observations.

### Fidelity of the HIV risk screening process

#### General communication

The majority of observed screenings (79%, n = 131) were conducted in private areas. Only partial privacy was maintained in the remaining cases; most of these screenings were conducted near roadsides and not in residences. CCW were observed to be asking questions and receiving answers in a non-judgmental manner in nearly all screenings (94%, n = 156). Of the 60 caregivers or children who raised questions or concerns as part of the screening, 77% (n = 46) were addressed well and 23% were addressed partially; there were no concerns observed to be unaddressed by CCW. Two referrals were issued for a health or social need unrelated to the HIV screening.

#### Tool completeness and variability

Among all observations, composite scores ranged from 10 to 49 out of a total possible score of 50 (Table 2). The overall median score for the observations was 42 (IQR 36–45), with a median screening tool score of 34 (IQR 28–36) out of 41 and a median general communication score (comprised of components described above) of 8 (IQR 8–9) out of 9 points. For CCW who contributed multiple observations (n = 27), a median composite score was calculated per CCW. The lowest score was 22 out of four observations conducted; the highest score was 46 (only one observation). Scores of screenings conducted with children < 10 years were slightly higher than those conducted with adolescents ≥ 10 years.

**Table 2:** Observation screening scores.

		Screening Tool Question Score	General Communication Score	Total Score
Total possible points		41	9	50
	N	Median (IQR), Range		
Observations	166	34.0 (28.0–36.0), 1.0–40.0	8.0 (8.0–9.0), 5.0–9.0	42.0 (36.0–45.0), 10.0–49.0
Average CCW score	32	34.3 (29.5–36.0), 16.0–39.0	8.0 (7.5–9.0), 6.0–9.0	42.3 (37.5–44.6), 22.3–46.0
**CCW by age of child screened**	N (%)			
< 10 years	81 (48.8)	36.0 (31.0–37.0), 6.0–40.0	9.0 (8.0–9.0), 5.0–9.0	43.0 (40.0–46.0), 12.0–49.0
≥ 10 years	85 (51.2)	32.0 (28.0–36.0), 1.0–39.0	8.0 (8.0–9.0), 5.0–9.0	40.0 (36.0–44.0), 10.0–47.0

**Fig 2:**
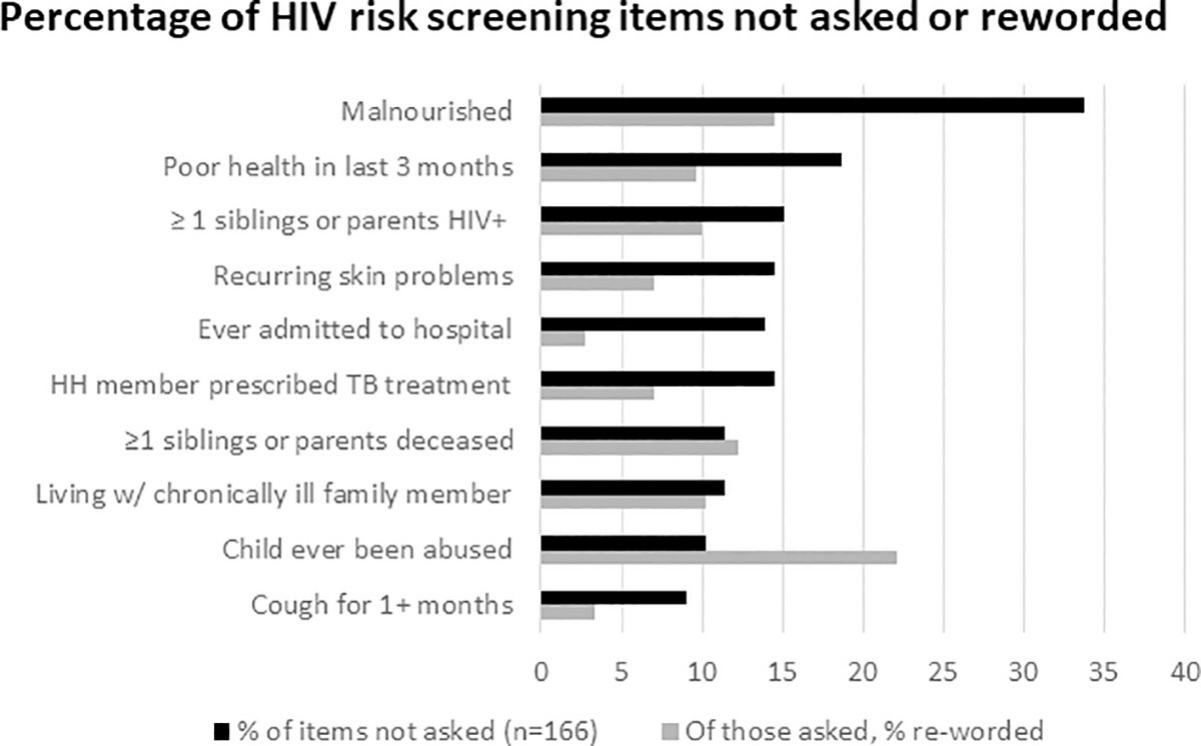
Percentage of HIV risk screening items not asked or reworded.

Of the 10 screening items to be asked to all beneficiaries 2–19 years of age, malnutrition, the most frequently skipped question, was not asked in about 34% of the observed screenings (Fig 2); 41% (n = 13) of CCW skipped this question during one or more screenings. Other items were skipped, ranging from 9% for ‘cough for >1 month,’ to 19% for ‘poor health in last three months’ of the observed screenings. The questions on sexual activity and pregnancy were only meant to be asked to adolescents ≥ 10 years of age. Of those adolescents observed (n = 85), 20% (n = 17) were not asked if they were sexually active and 45% (n = 38) were not asked if they were pregnant or had a child of her own; 31% (n = 10) and 41% (n = 13) of CCW respectively skipped these questions during one or more screenings. There were seven CCW who skipped all or nearly all questions at ≥ 1 screenings. On the other hand, 6% (n = 5) of children < 10 years and/or their caregivers were asked about sexual activity and 5% (n = 4) were also asked about pregnancy. Among those ≥ 10 years who were asked about sexual activity (n = 68), there was variation in how the question was asked, with 44% (n = 30) asking just the main question on sexual activity and 35% (n = 24) asking some combination of probing questions listed in the SOP with or without the main question.

According to the observers’ comments, some questions were not asked based on CCW knowledge or presumed knowledge of household members (e.g., pregnancy, having a child, death of family members, HIV status). In the majority of observations in which malnutrition was not asked, observers explained that the CCW often relied on their own visual assessment of the child to determine the answer to this question. In general, RAs indicated that CCW often completed answers on the tool without asking them aloud, particularly if the same caregiver had more than one child/adolescent who underwent screening. CCW were also observed asking the questions and immediately offering answers without waiting for the caregiver to respond. For three adolescents, CCW reported to RAs that the question on sexual activity was asked, but done out of earshot of others to respect adolescent privacy.

#### Screening tool perceptions and ease of use

In the focus group discussions, CCW generally reported feeling comfortable with the questions in the screening tool. CCW indicated that beneficiaries found the following questions most difficult to answer, starting with the highest number of CCW indicating beneficiary difficulty: adolescent being sexually active (n = 24 CCW), having a sibling or parent living with HIV (n = 18 CCW), and abuse of the child (n = 13 CCW). However, only a few CCW indicated that they had difficulties asking the questions. While most CCW did not recommend removing any questions from the tool, they described some of the screening items as containing direct, harsh language, which they characterized as socially inappropriate to ask. They described how they reworded such questions and suggested that the tool be revised to reflect ‘softer’ language. This issue emerged most often with the sexual activity risk item. In their FGD responses, CCW described multiple ways they asked this question, deviating from the tool and the probing questions in the SOP. Examples of CCW-reworded questions included ‘what is the name of your boyfriend?’ and ‘have you started love affairs?’ As described by one respondent,

If you ask him, ‘have you started having sex?’ It is harsh. But if you ask ‘have you started looking for girls,’ this one is not harsh, it is easy and you still mean the same thing. (CCW)

Rather than using these questions as a starting point or for probing when no answer is forthcoming, these reworded questions seemed to be used in place of more direct questions asking about sexual activity or condom use per the screening tool. Another reason for the variation was the familiarity that came with the CCW also being a member of the same community and someone who visits the household regularly.

There are other children who see you as a very weird person…’In our street, I always greet this person. Today he asks me about sex? Why is he disrespecting me?’ (CCW)

While some CCW felt adolescents may find it awkward to be asked these sensitive questions by someone known to them, most adolescent respondents indicated in the interviews that they found the questions normal and easy to answer. Only a few reported the sexual activity question was difficult to answer:

I felt a bit bad, she is an adult and a parent, and I felt that… there was a question she asked that I wasn’t free to answer immediately, that was why I found it hard, and then I came and answered later. (Adolescent)

Given these difficulties, CCW described creative ways of getting the adolescent to open up, such as sneaking away with him or her to ask privately or speaking in youthful rhetoric. In addition to asking the question, CCW also described in some cases thoroughly assessing other items, beyond simply asking the question. For instance, in an effort to determine if the child was in an abusive household, they would pull young children aside and ask them out of caregiver earshot, examine sleeping arrangements, and be vigilant to changes in mood or behavior across household visits.

### Acceptability of home screening and testing

Despite some of the challenges with individual screening questions, overall, caregivers and adolescents who participated in IDI found home-based risk screening acceptable (Table 3). Beneficiaries typically had positive or neutral feelings about the screening process; only two indicated that they were ‘shocked’ at first by the screening questions but came to understand the purpose with CCW explanation. For their part, CCW participating in FGD stressed the importance of establishing rapport and relationship building, with some beneficiaries expressing gratitude for their support. There were no negative consequences reported because of screening by beneficiaries. One CCW-identified challenge was that there was an expectation that beneficiaries should receive more support in return for registration in the program and for participating in quarterly home visits by the CCW. This expectation may have been born out of previous provisions to households (from this program or another) coupled with often impoverished living conditions.

**Table 3:** Agreement with statements on HIV risk screening and testing.

	Adolescents (n = 17)	Caregivers (n = 25)	Total (n = 42)
Statement–Screening, N (%)			
Overall, I am satisfied with the visit by the community worker in my home.	17 (100%)	25 (100%)	42 (100%)
I found the questions asked by the community worker about me/my children and the household easy to answer.	13 (77%)	25 (100%)	38 (91%)
I am satisfied with the time taken for these questions to be asked.	17 (100%)	25 (100%)	42 (100%)
The community worker addressed questions that I had and/or provided me with a referral for my health and other needs.*	12 (71%)	21 (84%)	33 (79%)
Someone I did not want to hear my responses to these questions may have overheard them.	0	1 (4%)	1 (2%)
Statement–HIV Testing, N (%)			
I am satisfied with the HIV testing by the research nurse in my home.	17 (100%)	25 (100%)	42 (100%)
I would (arrange for the children in my care to) present at a health facility for HIV testing if referred for testing by the community worker.	15 (88%)	24 (96%)	39 (93%)
I am satisfied with the time taken for HIV testing.	17 (100%)	25 (100%)	42 (100%)
I would prefer to be tested in the home instead of the health facility.	15 (88%)	24 (96%)	39 (93%)
Someone I did not want to be present during counseling or testing may have overheard or learned my (my children’s) status.	0	1 (4%)	1 (2%)

*Three adolescents and one caregiver responded with ‘no opinion,’ interpreted as they did not have questions or referral needs. Only two other ‘no opinion’ responses were provided: prefer to be tested in the home (1 caregiver) and somone may have overheard or learned my status (1 adolescent).

Similarly, caregiver and adolescent beneficiaries also found HTS in the home acceptable (Table 3). Adolescents and caregiver beneficiaries generally felt good about HIV testing in the home and learning their HIV status. Beneficiaries and CCW felt home testing should be ongoing, frequent, and extended to OVC caregivers and possibly the wider community as well. Commonly reported advantages of home testing over facility-based testing included no costs incurred for transport to the clinic, convenience, and timesaving, particularly for older caregivers and caregivers with many children, and confidentiality. This caregiver described stigma attached simply to attending the health facility with her children, which can be mitigated through home-based testing.

When you go [to the hospital], everyone will start looking at you and even when you go to the testing room they will start saying ‘it’s done, she has taken her children for testing, she has even transmitted it to her children.’ Meanwhile you just went there to check your health and to know about the health conditions of your children. (Caregiver, both age groups)

Most caregivers of children <10 years did not talk to them about their results, while all caregivers of children 10–17 years who were asked about disclosure discussed results with them. Some caregivers described using home testing as a platform to disclose HIV test results to their children and to discuss adopting healthy behaviors and avoiding risky ones. Several adolescents indicated that they felt it was important to know and share test results with their caregivers. Caregivers and adolescents reported that receiving negative test results motivated them to be healthy.

I felt happiness, because I wanted to know more about…my health status. But now, because I have tested, I am happy with the results I got. From the results I got, I will be ready for anything. I will be ready to protect myself against HIV infections. (Adolescent)

While respondents felt confidentiality was better protected at home, CCW described the potential for compromises in home settings, particularly in crowded tenant properties. When this seemed to be an issue, CCW reported that they moved to another location, closed off into one room, or deferred to the beneficiary’s preference. Only two breaches were described; in one case a father was present for post-test counseling against the adolescent’s wishes and in another, a tenant was present during screening and testing for a child, though not for delivering test results and post-test counseling. Several beneficiaries described that neighbors would come by during the CCW’s visit, ask questions about the nature of the visit, or gossip about the visitors and their purpose, but that they generally dismissed their questions with vague responses. CCW indicated that the CSOs they work for were known in communities to be associated with HIV issues, which seemed to fuel rumors about the HIV status of household members as well as the CCW themselves.

## Discussion

Among community workers conducting home visits, there was sub-optimal fidelity to administration of an HIV risk screening tool for children and adolescents registered in an OVC program. The position of CCW as community residents with possible prior knowledge of the family, frequency of visits to the household, and the phrasing of some of the screening items seemed to contribute to variable implementation of the tool. However, beneficiaries found home-based HIV risk screening and testing acceptable overall and they recommended its continuation and expansion.

Tool items were not consistently asked and some questions were often reworded; in some cases, questions were asked to the wrong age group. While there was variability, many of the same CCW were responsible for skipping questions in multiple screenings, with about 22% of CCW consistently not asking questions. While some questions could be justifiably skipped (e.g., a household member being prescribed TB treatment), the instructions were to screen each child by asking every question, so we evaluated according to this standard. The interviewer and setting can influence the fidelity of HIV risk screening implementation. CCW were members of the same community as the beneficiaries and have established or were working to establish relationships with these households. A familiar or close relationship could help or hinder tool administration and the elicitation of accurate responses.

These relationships could result in differences in how clinical or lay providers would administer the screening tool in facilities. For instance, CCW often skipped or reworded the sexual activity question directed towards adolescents. The establishment of trust between the CCW and beneficiaries is an advantage of the home-based approach and may have allowed for greater openness, particularly among adolescents. Some CCW also described creative approaches to obtaining answers for this question and variations on the question, which may not have prompted an accurate answer. On the other hand, there may not be the same level of embarrassment in a facility setting with a provider, which could allow for a more straightforward, objective screening process. However, sensitive screening questions and discussions around sexual history have presented difficulties in clinic settings as well [5, 23]. A question on STI symptoms was excluded from the validation phase of a screening tool evaluation in Zimbabwe because it was felt to be inappropriate for providers to ask in a population of 6–15 year olds [16, 17].

There are limited existing data on the fidelity with which screening tools are implemented; however, the findings from this study suggest that the predictive value of the screening tool (findings published separately) may depend not only on the risk factors themselves, but also on how they are assessed. The most frequently skipped items in these observations was malnutrition and poor health in the last three months in 34% and 19% of observed screenings, respectively. A study from Uganda showed that malnutrition and recurrent illness were among the most highly-predictive factors for HIV positive status among newly diagnosed children less than 15 years of age [24].

Tool sensitivity may be optimized or compromised in programs depending on how the tool is administered to beneficiaries. One way to improve tool implementation is to reinforce standardization of the tool and greater adherence to procedures through further training and mentorship. This could include integrating observation by supervisors into the screening process at household level. This study’s findings suggest changing the wording of the screening items, to address the comfort level with asking and answering the questions and to provide greater clarity on what is being asked. Community input could inform phrasing and translation of screening items to standardize language in a culturally-appropriate way. Questions should be worded and asked in ways that help to ensure comfort of the respondent and encourage honest answers, but this should not be entirely improvised or up to individual screeners to revise questions. In consideration of these study findings, reviewing questions with community members as well as experts and making revisions to screening items and the standard operating procedures where needed, could improve cultural appropriateness, but also accuracy and consistency of the tool and its administration to the beneficiary population.

Despite variable implementation of the HIV risk screening tool, CCW and beneficiaries generally found the screening approach in the home setting acceptable. Other studies have demonstrated that lay staff can administer HIV risk screening tools in facility and community settings, though these did not include process evaluations to assess fidelity [17, 22]. Training community health workers to conduct home-based screenings for non-communicable diseases (e.g. hypertension, diabetes, cancer) has also been found to be feasible, though some community health workers were overburdened with providing various other services [25, 26].

Although home-based HIV testing after HIV risk screening was introduced as part of the validation study procedures, we found widespread support for a combined home-based screening/testing service. Caregivers expressed sentiments similar to guardians in one survey from Zimbabwe, the majority of whom reported that they would be happier knowing their child’s HIV status, despite fears around a possible positive diagnosis [27]. Given that obstacles to accessing facility-based HTS may be exacerbated for the OVC population and their caregivers, home-based HIV testing should be considered. A home visiting program in South Africa doubled the odds that an orphan would receive HIV testing [21]. Other studies and pooled analyses have found high acceptability of home testing, with testing acceptance rates around 85%, though these did not focus specifically on vulnerable populations [28, 29]. HIV self-testing may be another way to help improve access to testing for adolescents, leveraging existing community support structures for assisted home-based testing and follow-up [30].

Respondents described a number of barriers to facility-based testing and indicated their preference for testing in the home. In particular, beneficiaries were concerned about privacy at the clinic, often fearing others could draw the conclusion that they were positive even if just going for an HIV test. Testing location preferences in the literature are mixed. In one study, 88% of caregivers preferred home-based testing [31]. Another study found 75% of caregiver index clients chose clinic based testing for their children; however, those who selected community-based testing were nearly two times more likely to actually receive the test than those opting for the clinic [32]. In our study, many respondents also reported that they would go to the facility if they were referred by the CCW. Even though none of the children or adolescents were found to be HIV-positive in this sample, receiving a negative HIV test result was an opportunity for some to recommit to, or discuss with one’s family, the adoption of healthy behaviors. In addition to emphasizing risk reduction, simply undergoing HIV testing can increase the likelihood of subsequent uptake of testing and possibly reduce the likelihood of future HIV acquisition [33, 34].

This study had some notable limitations. The observation component was conducted with a relatively small number of CCW, many of whom contributed data for multiple observed screenings. Moreover, the observation approach may have influenced CCW behavior. Observations could have made them more aware or nervous, providing an underestimated picture of performance or resulting in social desirability bias, possibly overestimating their performance. All observational approaches have their limitations, but use of multiple methods can be used to triangulate findings, such as structured exit interviews with beneficiaries or other qualitative data collection to help contextualize observation findings as we have done in this study. The perceptions of home-based screening and testing were generally positive among adolescents and caregivers, which may have been due to the fact that we were not able to include any interviews from those who had refused testing or who had tested HIV-positive. Future studies should consider specifically targeting participants who test HIV-positive to assess acceptability of home-based HIV risk screening and HIV testing among this group.

## Conclusion

Validation studies have identified high performing risk screening items, but there are minimal published data on the process of screening tool administration and the extent to which questions are asked with fidelity. We found sub-optimal fidelity to administration of the screening tool by CCW in the home environment to children and adolescents enrolled in an OVC program. Improvements to questions and their delivery could strengthen tool performance and help improve HIV case finding using a targeted testing approach. Continued mentorship is also needed to improve the assessment of critical but sensitive information in this population, such as child abuse, sexual activity and pregnancy. Overall, home-based HIV risk screening and testing was acceptable in this population. Coupling home-based testing with screening could help ensure testing services are accessible to those who may not know their status and serve as a platform to promote healthy behaviors for those with limited interactions with the health system.
